# Surgical Stress Abrogates Pre-Existing Protective T Cell Mediated Anti-Tumor Immunity Leading to Postoperative Cancer Recurrence

**DOI:** 10.1371/journal.pone.0155947

**Published:** 2016-05-19

**Authors:** Abhirami A. Ananth, Lee-Hwa Tai, Casey Lansdell, Almohanad A. Alkayyal, Katherine E. Baxter, Leonard Angka, Jiqing Zhang, Christiano Tanese de Souza, Kyle B. Stephenson, Kelley Parato, Jonathan L. Bramson, John C. Bell, Brian D. Lichty, Rebecca C. Auer

**Affiliations:** 1 Center for Innovative Cancer Research, Ottawa Hospital Research Institute, Ottawa, ON, Canada; 2 Department of Biochemistry, Microbiology and Immunology, University of Ottawa, Ottawa, ON, Canada; 3 Department of Medical Laboratory Technology, University of Tabuk, Tabuk, Saudi Arabia; 4 Department of Cellular and Molecular Medicine, University of Ottawa, Ottawa, ON, Canada; 5 Department of Neurosurgery, The Second Hospital of Shandong University, Shandong, China; 6 McMaster Immunology Research Centre, McMaster University, Hamilton, ON, Canada; 7 Department of Surgery, University of Ottawa, Ottawa, ON, Canada; Exploratory Oncology Research & Clinical Trial Center, National Cancer Center, JAPAN

## Abstract

Anti-tumor CD8^+^ T cells are a key determinant for overall survival in patients following surgical resection for solid malignancies. Using a mouse model of cancer vaccination (adenovirus expressing melanoma tumor-associated antigen (TAA)—dopachrome tautomerase (AdDCT) and resection resulting in major surgical stress (abdominal nephrectomy), we demonstrate that surgical stress results in a reduction in the number of CD8^+^ T cell that produce cytokines (IFNγ, TNFα, Granzyme B) in response to TAA. This effect is secondary to both reduced proliferation and impaired T cell function following antigen binding. In a prophylactic model, surgical stress completely abrogates tumor protection conferred by vaccination in the immediate postoperative period. In a clinically relevant surgical resection model, vaccinated mice undergoing a positive margin resection with surgical stress had decreased survival compared to mice with positive margin resection alone. Preoperative immunotherapy with IFNα significantly extends survival in surgically stressed mice. Importantly, myeloid derived suppressor cell (MDSC) population numbers and functional impairment of TAA-specific CD8^+^ T cell were altered in surgically stressed mice. Our observations suggest that cancer progression may result from surgery-induced suppression of tumor-specific CD8^+^ T cells. Preoperative immunotherapies aimed at targeting the prometastatic effects of cancer surgery will reduce recurrence and improve survival in cancer surgery patients.

## Introduction

The importance of immune surveillance in controlling outgrowth of malignant cells has been known for decades. The principle that naturally occurring T cells with antitumor potential exist in human cancer has led to the emerging field of cancer immunotherapy. Tumors themselves, however, are able to hijack the immune system to suppress immune control, creating a favourable environment, not only for the primary but also for metastatic outgrowth. The latter process has been termed "systemic instigation” [1]. Understanding this immunosuppressive tumor environment and reversing it with targeted agents is proving to be a highly successful treatment strategy [2].

Surgical resection is the mainstay of treatment for curative intent in solid malignancies. Even with complete resection, many patients develop local recurrence or metastases and ultimately die of their disease [3–6]. Accumulating evidence shows an association between the presence of CD8^+^ effector T cells and improved prognosis and survival in a number of solid malignancies, including breast, ovarian, colorectal, renal cell carcinoma, and malignant melanoma [7–9].

This association suggests that the presence of a robust anti-tumor immune response, in particular anti-tumor CD8^+^ effector T cells, can prevent or eliminate minimal residual disease or micrometastases following complete surgical resection of the primary tumor.

Our group and others have demonstrated that the immediate postoperative period is a uniquely susceptible time for the development of cancer recurrence metastases [3, 4, 10]. There are a number of mechanisms proposed to explain this effect including dissemination of tumor cells into the circulation [11], local and systemic release of pro-angiogenic and mitogenic mediators (epidermal growth factor and vascular endothelial growth factor) [3, 10, 12] and profound inhibition of cell-mediated immunity [3, 10]. We have previously implicated surgery-induced suppression of natural killer (NK) cells in the development of postoperative metastases [3, 4] in a validated animal model of surgical stress and metastases [13].

While previous studies have reported a reduction in CD8^+^ T cells and decreased cytokine secretion in response to non-specific stimulation [14–17], none have explored the nature of the effects of surgical stress on antigen specific CD8^+^ T cell responses nor have they demonstrated the abrogation of a pre-existing protective anti-tumor CD8^+^ T cell response. In this manuscript, we explore the effects of surgical stress on a protective CD8^+^ effector T cells mediated anti-tumor immunity. To accomplish this, we use a viral-vector based tumor vaccine called AdDCT. This is a replication-incompetent E1/E3-deleted human type 5 Adenovirus (Ad) that expresses the full-length human Dopachrome Tautomerase (hDCT) gene [18] as a tumor-associated antigen (TAA). It has been previously shown that prophylactic AdDCT vaccination can confer protection against subcutaneously (sc) implanted melanoma in a CD8^+^ T cell-dependent manner [18–21]. We provide preclinical *in vivo* results that clearly indicate that major surgery can significantly attenuate a pre-existing protective T cell immune response following cancer vaccination and provide the novel use of preoperative immunotherapy to reverse this effect.

## Materials and Methods

### Mice

C57BL/6, BALB/c, and CD1-nude mice were purchased from The Jackson Laboratory (Bar Harbor). Animals were housed in pathogen-free conditions and all studies performed were in accordance with institutional guidelines at the Animal Care Veterinary Service facility of the University of Ottawa (Ontario). The Canadian Council on Animal Care and the Animal Care Committee of the University of Ottawa approved this study.

### Generation of surgical stress

Routine perioperative care (including anesthesia and pain management) and surgical stress were performed as previously described [3, 13]. Briefly, mice were subjected to 2.5% isofluorane (Baxter Corp.) for induction and maintenance of anesthesia. Surgical stress was induced in mice by an abdominal laparotomy (3-cm midline incision) and left nephrectomy (Nx). All animals undergoing surgical procedures will receive pre and post-operative buprenorphine injections (0.05mg/kg) administered SC. Preoperatively, mice are injected SC 1 hour before surgery. Postoperatively, mice are injected SC every 8 hours for 2 days. Body weight and other aspects of animal wellness are recorded daily following surgery (S1 Table). Blood data is not collected. Mice were euthanized by intraperitoneal (ip) injection of Pentobarbital Sodium (65mg/kg).

### Cell lines

B16F10LacZ melanoma cell line was obtained from Dr. K. Graham (London Health Sciences, Ontario, originally from American Type Culture Collection) and maintained in complete DMEM. Cells were resuspended in DMEM without serum for intravenous (iv) injection through the lateral tail vein. Tumor cells at >95% viability were injected in a 0.1ml volume/mouse.

### Vaccination of mice

Anesthetized mice were vaccinated with 1x10^6^ plaque forming units (PFU) of AdDCT (reconstituted in 100μl PBS) or PBS alone by intramuscular (IM) injection into each thigh (50μl per thigh).

### Prophylactic vaccination tumor models

Mice were vaccinated with AdDCT or PBS on day 0. On day 7, mice were injected with tumor cells either sc or iv preceding surgery. Sc melanoma tumors were established by injecting 3x10^5^ B16F10lacZ cells in serum-free media on the right hind flank; tumor size was measured twice a week and tumor volumes were calculated using the formula 1/2(L×W^2^). Mice were determined to have reached endpoint when tumor volumes reached 1600mm^3^. Systemic dissemination models were seeded at 1x10^6^ B16F10lacZ cells through tail vein iv administration. Tumor-bearing lungs were stained with X-gal to visualize tumor micrometastases as previously described [3].

### Therapeutic vaccination tumor model

Mice were injected sc with 1x10^5^ B16F10lacZ cells in serum-free media on the right hind flank on day 0. On day 7, mice were treated with AdDCT or PBS at the indicated dose (pfu). On day 14, sc tumors were resected with a 2x2 mm positive margin, with half receiving additional surgical stress in the form of abdominal laparotomy and left nephrectomy. Tumor size was measured twice a week and tumor volumes were calculated using the formula 1/2(L×W^2^). Mice were determined to have reached endpoint when tumor volumes reached 1600mm^3^. For preoperative IFNα treatment (R&D Systems), mice received 1 high dose (10000 IU) at day 10 and 3 low doses (1000 IU) on days 11 through 13.

### Peptides

The immunodominant peptide from DCT that binds to H-2K^b^ (DCT_180–188_, SVYDFFVWL; shared by human and murine DCT) was synthesized by Biomer Technology (Pleasanton, California).

### Flow cytometry

For extracellular staining, splenocytes were incubated for 30 minutes at 4°C in the dark with fluorochrome- conjugated antibodies (diluted 1:100) specific for cell surface antigens and fixed with 1% PFA. The following antibodies were used: CD3-PerCP (Biolegend), CD8α- FITC (BD Biosciences), CD4-PE (BD Pharmingen), Gr-1-FITC (eBioscience), CD25-FITC (Biolegend), FoxP3-APC (eBioscience), CD11c-PeCy7 (eBioscience) and CD11b-PE (eBioscience). For intracellular staining, 1-2x10^6^ splenocytes were stimulated with DCT peptides (2μg/ml) in the presence of brefeldin A (GolgiPlug; BD Pharmingen, 1μg/ml added after 1.5 hours of incubation). Following 6 hours of incubation, cells were treated with anti-CD16/CD32 and cell surface antigens were labeled with fluorochrome-conjugated antibodies. Cells were then permeabilized and fixed with Cytofix/Cytoperm (BD Pharmingen) and stained for intracellular cytokines: IFNγ-PE (BD Biosciences), TNFα-PeCy7 (Biolegend) and Granzyme B-APC (eBioscience). Data was acquired using a Cyan-ADP 9 flow cytometer with Summit 4.3 software (Beckman Coulter) and analyzed with Kaluza 1.2 software (Beckman Coulter).

### DCT-specific MHCI tetramer analysis and ELISpot

Splenocytes were treated with anti-CD16/CD32 and labelled with PE-conjugated DCT_180-188_ peptide-loaded MHC-I tetramer (Baylor College), PerCP-CD3, and FITC-CD8α (eBioscience) for 30 minutes in FACS buffer (1% PBS, 5% fetal calf serum, 2.5% NaZ). For ELISpot analyses, the exact number of DCT-MHC-I tetramer^+^/CD8^+^ T cells were calculated from flow cytometry proportion results and added to an IFNγ ELISpot following manufacturer’s instructions (Mabtech).

### T cell apoptosis assay

Splenocytes were treated with anti-CD16/CD32 and cell surface antigens were labeled with PE-CD3 and FITC-CD8α (eBioscience). Cells were then labelled with APC-AnnexinV and 7-AAD (BD Pharmingen) for 15 minutes and data was acquired on the Cyan-ADP (Beckman Coulter) within an hour.

### BrdU T cell proliferation assay

Splenocytes were restimulated in vitro with peptides (2μg/ml) at 37°C in the presence of brefeldin A (GolgiPlug; BD Pharmingen, 1μg/ml added after 1.5 hours of incubation). After 3.5 hours of incubation, cells were labelled with BrdU (BD Pharmingen) and incubated for an additional hour. After 6 hours of incubation, cells were treated with anti-CD16/CD32 and cell surface antigens were labeled with PerCP-CD3 and PE-CD8α. Following manufacturer’s instructions, cells were treated through a series of permeabilizations and fixations and stained for intracellular FITC-BrdU.

### MDSC-T cell suppression assay

T cells were isolated from the spleens of mice that received AdDCT vaccination 7 days prior to harvest. MDSCs were isolated from the spleens of control and surgically stressed mice (18 hours post-surgery). T cells were positively selected for using CD90.2 MicroBeads (Miltenyi Biotec) and MDSC were positively selected for using the Myeloid-Derived Suppressor Cell Isolation Kit (Miltenyi Biotec), both following manufacturer’s protocols. Cells were resuspended at 1×10^6^ cells/50μL cRPMI and cocultured at various MDSC:T ratios (0.25:1, 0.5:1, 1:1). CD3/CD28 Dynabeads (Life Technologies) were added to the culture in a 1:1 bead- to- cell ratio in addition to 30 U/mL of mouse recombinant interleukin-2 as per manufacturer’s recommendation. Cells were then cultured for 4 days in a CO_2_ incubator at 37°C. After 4 days, cells were transferred to a V- bottom 96-well plate for re- stimulation with DCT peptide using the protocol listed above for intracellular staining.

### Statistical analysis

One-way analysis of variance (ANOVA) and the Bonferonni post hoc test were performed for all data. A P-value <0.05 was considered significant. Statistical significance of Kaplan-Meier survival curves were determined using log-rank tests.

## Results

### Surgical stress impairs antitumor immunity resulting in lung metastasis and subcutaneous tumor outgrowth

**Fig 1:**
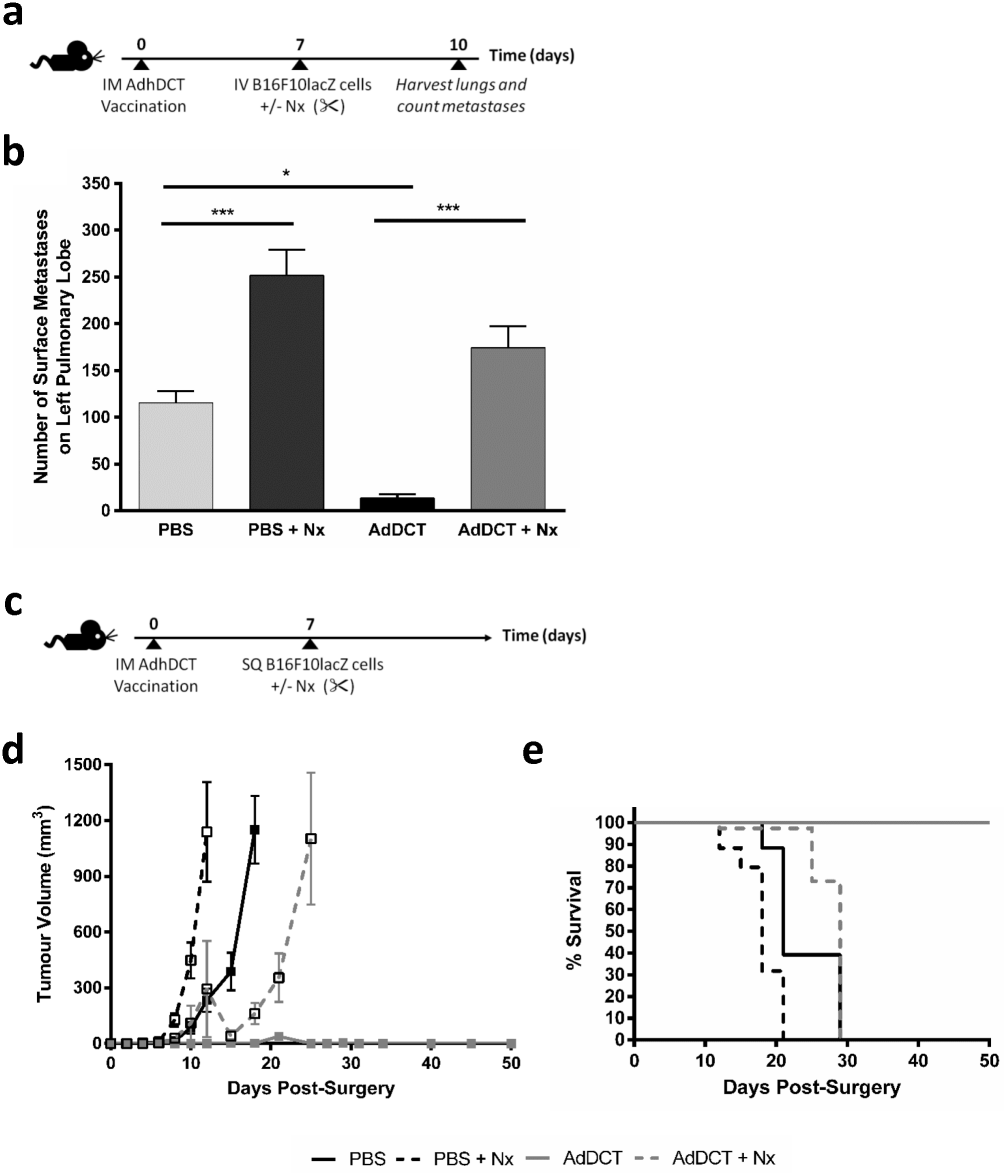
Surgical stress impairs anti-tumor immunity resulting in lung metastasis and subcutaneous tumor growth. **(a)** B6 mice were administered with neoadjuvant AdDCT at 1×10^7^ pfu. On day 7, mice were challenged iv with 3x10^5^ of B16F10lacZ cells in order to establish syngeneic lung melanoma metastases, and then underwent abdominal laparatomy and nephrectomy (Nx) or no surgery. **(b)** Lungs extracted at day 10 (3 days following tumor cell inoculation) from PBS, Nx, AdDCT, AdDCT+Nx and quantified by X-Gal staining. N = 5/group. **(c)** B6 mice were vaccinated with AdDCT, then challenged on day 7 with sc 1x10^6^ B16F10lacZ cells, then underwent surgery or no surgery. **(d)** Tumor volume of treated B16F10lacZ sc tumor bearing mice was monitored daily. **(e)** Survival of treated B16F10lacZ sc tumor-bearing mice are shown in Kaplan-Meier curves. Percentage of living mice is indicated. N = 7-8/group, (*P<0.05, ***P<0.001).

To determine the impact of surgical stress on TAA-specific T cells, we developed an AdDCT vaccination surgery model. Using our established B16F10lacZ melanoma lung metastasis surgery model [3, 13], we administered neoadjuvant AdDCT followed 7 days later with tumor inoculation by iv injection and major surgery with a laparotomy and nephrectomy (Fig 1a). Vaccination with 1x10^6^ pfu of AdDCT conferred a 3-fold decrease in metastases compared to PBS treated control mice. However, vaccinated mice subjected to major surgery demonstrated a 5-fold increase in metastases compared to vaccination alone (Fig 1b). This suggests that surgery attenuates the antitumor immunity conferred by AdDCT vaccination.

It has been previously shown that iv injected melanoma cells are more susceptible to NK cell-mediated lysis in comparison to sc melanoma, in large part due to differences in tumor microenvironment and immune cell proportions [22, 23]. To rule out a mediating role for NK cells, we performed the tumor challenge using a sc injection of B16F10lacZ tumor cells in the hind flank (Fig 1c). In AdDCT-vaccinated mice who were not subjected to surgical stress, 100% rejected a subsequent tumor challenge whereas all of the mice who underwent a major surgery at the time of tumor challenge developed flank tumors at a rate similar to the control un-vaccinated mice (Fig 1d and 1e). In addition, we observed the attenuation of pre-existing anti-tumor immunity by surgical stress in a CT26 colorectal cancer model (S1 Fig), suggesting that surgery-induced dysfunction of tumor-specific immunity is not exclusive to our melanoma model. Taken together, these results suggest that surgical stress in AdDCT-vaccinated mice abrogates a protective anti-tumor immune response.

### Surgery induced abrogation of protection conferred by AdDCT vaccination is dependent on CD3^+^ T cell

**Fig 2:**
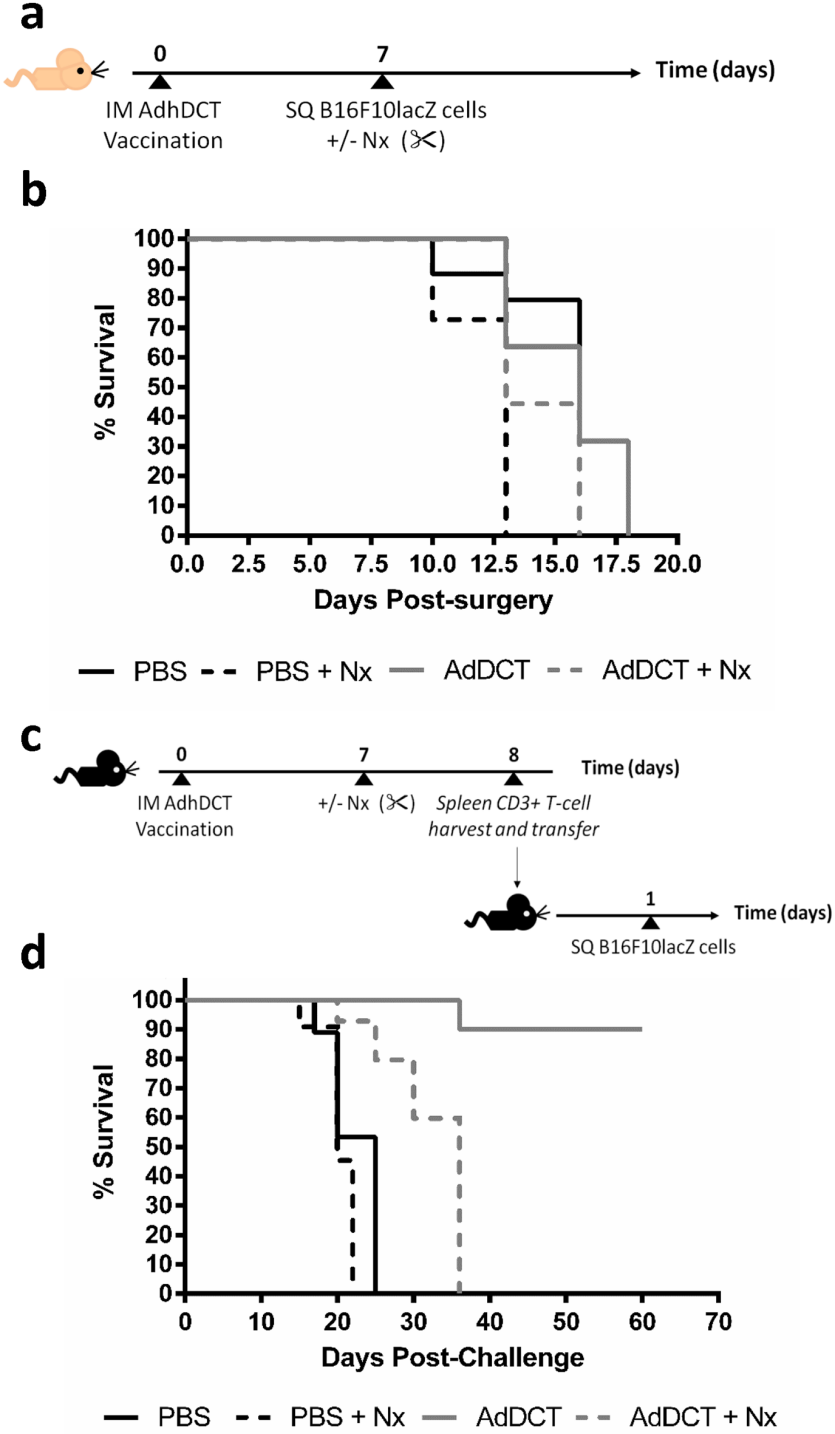
Surgery-induced abrogation of protection conferred by AdDCT vaccination is dependent on CD3^+^ T cells. **(a)** CD-1 nude mice were vaccinated with 1×10^7^ pfu AdDCT. On day 7, mice were challenged with sc B16F10lacZ tumors and then underwent surgery or no surgery. **(b)** Survival of treated B16F10lacZ tumor-bearing CD-1 nude mice are shown in Kaplan-Meier curves. Percentage of living mice is indicated. N = 7-8/group. **(c)** B6 mice were vaccinated with 1x10^7^ pfu AdDCT and at day 7, mice underwent surgery or no surgery. At day 8, spleen CD3^+^ T cells were isolated and transferred to naive recipient B6 mice. At day 9, recipient mice were challenged with sc B16F10lacZ tumors. **(d)** Survival of treated B16F10lacZ tumor-bearing mice are shown in Kaplan-Meier cures. Percentage of living mice is indicated. N = 7-8/group.

To demonstrate a mediating role for the adaptive immune system in surgery-induced impairment of protective AdDCT vaccination, we repeated the sc tumor challenge model in CD-1 nude mice that are deficient in T and B cells, but retain NK cell function [24] (Fig 2a). In nude mice, AdDCT vaccination did not protect mice form a tumor challenge and surgical stress did not result in a significant differences in survival in AdDCT-vaccinated mice (Fig 2b). This suggests that the adaptive immune system is essential for the protective effects of AdDCT vaccination and the negative effects of surgical stress on tumor outgrowth in our model. To establish T cells as the mediator for both tumor protection following AdDCT-vaccination and for susceptibility to tumor growth following major surgery, we adoptively transferred 1.0x10^7^ purified splenic CD3^+^ T cells from AdDCT-vaccinated mice and from surgically stressed AdDCT-vaccinated mice into naïve recipient mice. 1 day following T cell transfer, recipient mice were challenged with sc B16F10lacZ tumors (Fig 2c). Survival was monitored over time. The mice that received T cells from vaccinated donors were 90% protected from the B16 tumor challenge, while those that received the same number of T cells from surgically stressed vaccinated donors all developed progressive flank tumors (Fig 2d). By transfering surgically stressed T cells and recreating the effect of surgery on protective vaccination, we established that the detrimental effect of surgery on AdDCT vaccination is T cell mediated.

### Surgical stress results in a decrease in CD8^+^ T cell cytokine production in response to TAA

**Fig 3:**
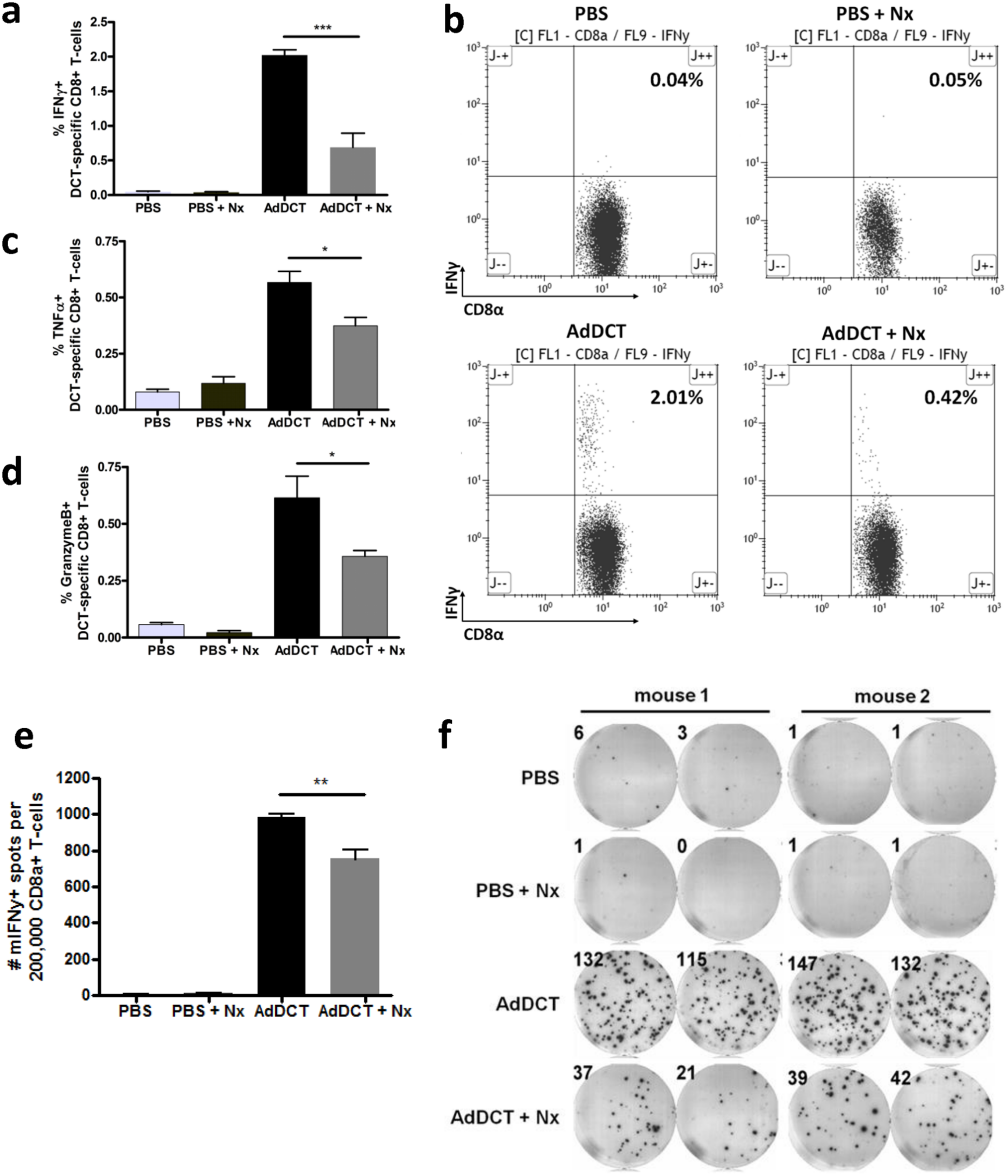
Surgical stress results in a decrease in CD8^+^ T cell cytokine production in response to TAA. B6 mice received neoadjuvant vaccination with 1×10^7^ pfu AdDCT. At day 7, mice were challenged iv with 3x10^5^ of B16F10lacZ cells in order to establish syngeneic lung melanoma metastases, and then underwent surgery or no surgery. At day 8, mice were sacrificed and underwent spleen immune cell assessment. Percentage of DCT-specific **(a)** IFNγ^+^, **(c)** TNFα^+^, **(d)** Granzyme B^+^ CD8^+^ T cells reacting to DCT_180-188_ peptide exposure. **(b)** Representative flow cytometry dot plots of DCT-specific IFNγ^+^/CD8^+^ T-cells reacting to DCT_180-188_ peptide exposure. **(e)** Quantification of SFU in IFNγ ELISpot assay. **(f)** Corresponding representative images of IFNγ ELISpot assay of CD8^+^ T-reacting to DCT_180-188_ peptide exposure. (*P<0.05, **P<0.01, ***P<0.001).

Since surgical stress resulted in attenuation of T cell mediated clearance of tumor cells *in vivo*, we questioned whether AdDCT induced T cell cytokine production in response to TAA (DCT) is affected by surgery. Using the same model of neoadjuvant AdDCT vaccination followed by tumor inoculation and major surgical stress 7 days later, we evaluated cytokine secretion in DCT-specific CD8^+^ T-cells isolated from the spleen at 18 hours following surgery. We observed a significant decrease in the proportions of CD8^+^ T cells secreting IFNγ (Fig 3a and 3b), TNFα (Fig 3c) and Granzyme B (Fig 3d) in response to DCT peptide stimulation and a reduction in the number of CD8^+^ T cells secreting IFNγ in response to non-specific stimulation with PMA and Ionomycin (S2 Fig). Furthermore, we performed an IFNγ ELISpot assay to corroborate our flow cytometry results and found a similar attenuation in the number of DCT-specific CD8^+^ T cells producing IFNγ following major surgery (Fig 3e and 3f). This data suggests that surgical stress results in a decrease in the number of TAA-specific CD8^+^ T cell secreting cytokines upon DCT peptide stimulation.

### Surgically stressed T cells display reduced cytokine secretion, proliferation and tumor infiltration in response to TAA

**Fig 4:**
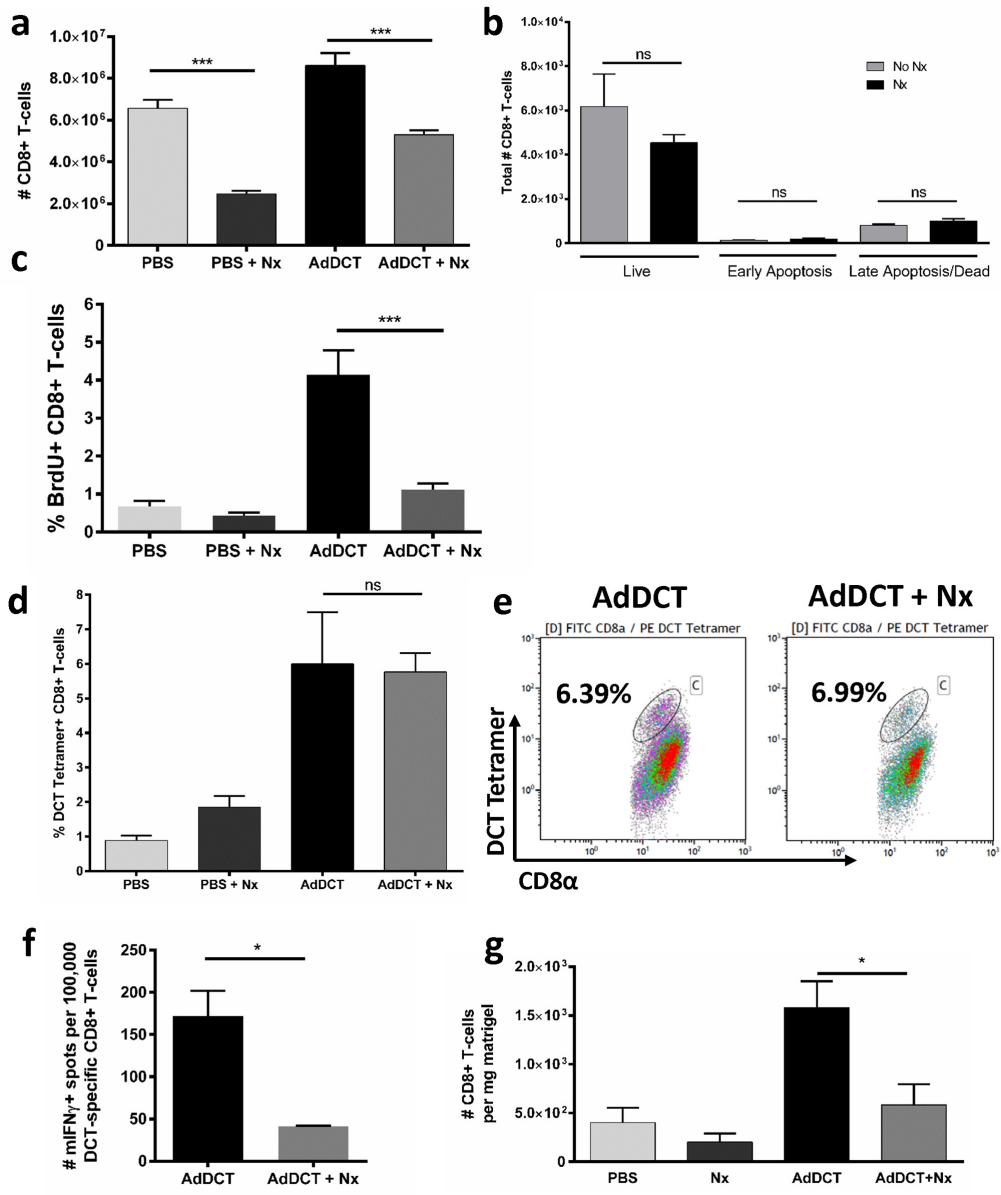
Surgically stressed T cells display reduced cytokine secretion, proliferation and tumor infiltration in response to TAA. B6 mice received neoadjuvant vaccination with 1×10^7^ pfu AdDCT. At day 7, mice were challenged iv with 3x10^5^ of B16F10lacZ cells in order to establish syngeneic lung melanoma metastases, and then underwent surgery or no surgery. At day 8, mice were sacrificed and underwent spleen immune cell assessment. **(a)** Total number of CD8^+^ T cells, **(b)** total number of Annexin V^-^/7-AAD^-^/CD8^+^ T (live), Annexin V^+^/7-AAD^-^/CD8^+^ T (early apoptosis), Annexin V^+^/7-AAD^+^/CD8^+^ T (late apoptosis) cells, **(c)** percentage of BrdU^+^/CD8^+^ T cells, **(d)** percentage and **(e)** representative dot plot of DCT-tetramer^+^/CD8^+^ T cells reacting to DCT_180-188_ peptide exposure, **(f)** quantification of SFU from DCT-tetramer^+^/CD8^+^ T cells reacting to DCT_180-188_ peptide exposure in IFNγ ELISpot assay. **(g)** Total number of CD8^+^/CD3^+^ T cells per mg of tumor from B6 mice challenged with sc B16F10lacZ tumors mixed with matrigel on day 7. On day 10, matrigel plugs were removed and assessed for immune cell infiltration by flow cytometry. N = 4-6/group. (*P<0.05, ***P<0.001).

Previous reports have demonstrated a global reduction in CD8^+^ T cells following surgery [10, 14]. Using our model of AdDCT vaccination followed by major surgery 7 days later, we also demonstrated a reduction in global numbers of CD8^+^ T cells isolated from the spleen 18 hours following surgery (Fig 4a). This reduction was not secondary to increased cell death, as measured by AnnexinV and 7AAD staining (Fig 4b), but was associated with a reduction in T cell proliferation, as measured by BrdU incorporation following DCT-peptide stimulation (Fig 4c). We next sought to determine whether the decreased number of CD8^+^ T cells secreting cytokine in response to DCT was secondary to a decrease in the proportion of DCT-specific CD8^+^ T cells (i.e. T cells capable of binding DCT) or a functional impairment of cytokine secretion following DCT-stimulation. Using an MHC class I DCT-specific tetramer, we demonstrated no reduction in the proportion of DCT-specific CD8^+^ T cells following surgical stress in vaccinated mice (Fig 4d and 4e). In order to confirm that DCT-specific CD8^+^ T cells were functionally impaired following surgery, we plated equal numbers of DCT tetramer-binding CD8^+^ T cells in an ELISpot following DCT peptide stimulation and observed a significant reduction in IFNγ secretion (Fig 4f). Additionally, we assessed the kinetics of IFNγ expression in DCT-specific CD8^+^ T cells at various time points following vaccination and surgery and observed a recovery period of T cell functionality between post-operative day (POD) 7 and POD 28 (S3a and S3b Fig). The observed recovery of T cell function at POD 28 also correlates with an antitumor immune response as surgery-recovered mice were able to reject a B16lacZ sc tumor challenge with equal efficacy as no surgery controls (S3c Fig). Finally we evaluated whether CD8^+^ T cell migration into the tumor was affected by surgical stress. To address this, we vaccinated mice with AdDCT followed 7 days later by sc injection of tumor cells mixed with a matrigel plug and major surgery. Matrigel plugs were assessed for infiltrating lymphocytes 3 days following implantation. Our data demonstrates that CD8^+^ T cells were decreased by half in the tumors of surgically stressed mice, indicating that surgery affects tumor immune infiltration (Fig 4g). Taken together, these results suggest that major surgery results in a global reduction in CD8^+^ T cells which is not secondary to increased cell death, but is associated with inhibition of proliferation. The proportion of DCT-specific T cells is not reduced relative to the global number, but the remaining DCT-specific T cells are dysfunctional in their ability to secrete IFNγ in response to tumor antigen. Finally, CD8^+^ T cell infiltration of tumors is also impaired following surgical stress.

### Surgery impairs vaccine function in a therapeutic B16 model of minimal residual disease (MRD) and can be partially rescued with perioperative IFNα

**Fig 5:**
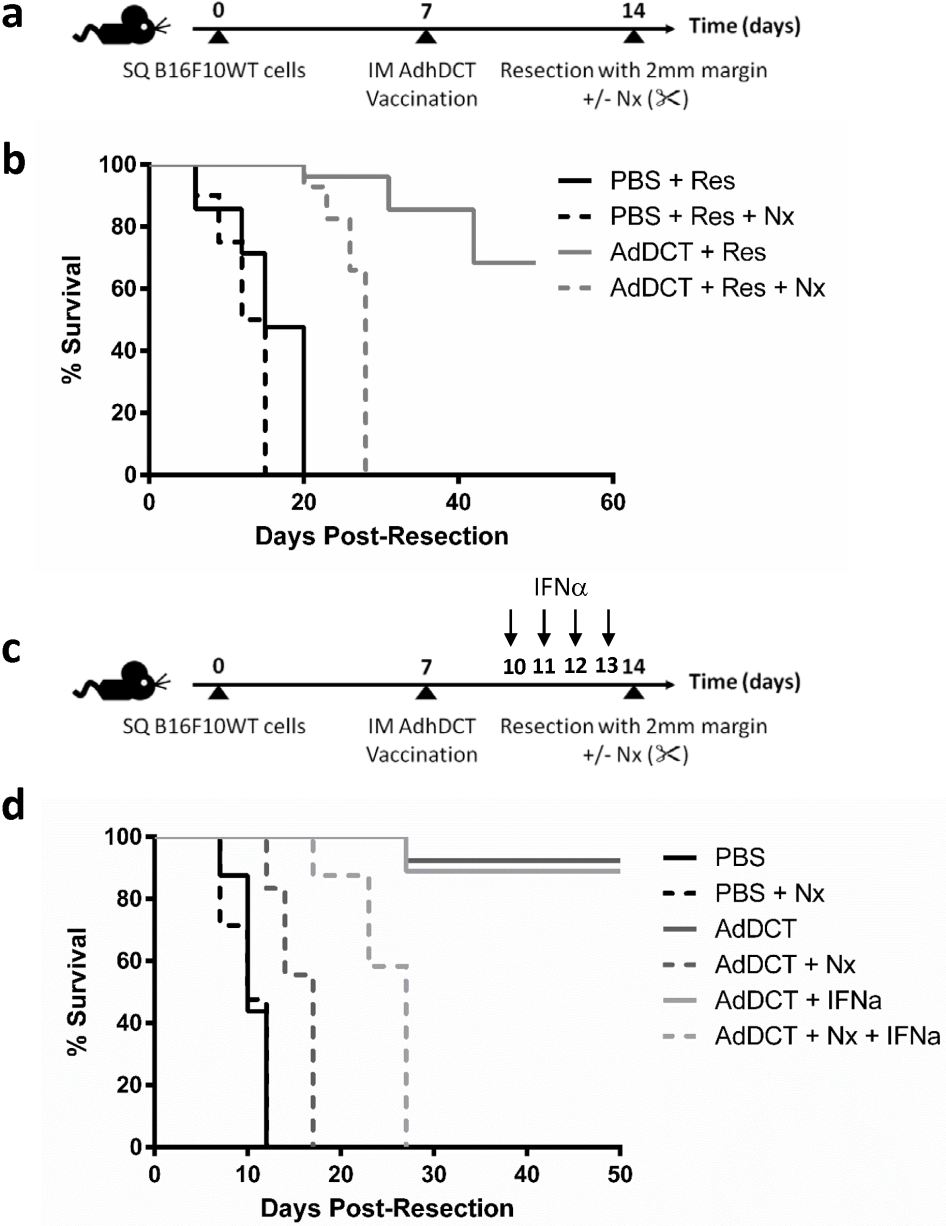
Surgery impairs vaccine function in a therapeutic B16 melanoma model of minimal residual disease and can be rescued with preoperative IFNα. **(a)** B6 mice were injected sc with 1x10^6^ B16F10lacZ cells. At day 7, mice were vaccinated with AdDCT. At day 14, tumors were resected (Res) with a 2mm margin +/- surgical stress (Res+Nx). **(b)** Survival of treated B16F10lacZ tumor-bearing mice are shown in Kaplan-Meier curves. N = 7-8/group. **c)** Preoperative treatment at day 10 with 1 high dose (10,000 IU/mouse) and at days 11 through 13 with 3 low doses (1000 IU/mouse) of recombinant mIFNα in MRD vaccination model. Survival of treated B16F10lacZ tumor-bearing mice are shown in Kaplan-Meier curves. N = 5-6/group.

Given the limitations of a prophylactic model of cancer vaccination, we developed a clinically relevant therapeutic B16 melanoma model of minimal residual disease (MRD) (Fig 5a). In this model, all mice had flank tumors implanted on day 0 followed by AdDCT vaccination on day 7, resection of their primary tumor with a 2 mm positive margin on day 14 to simulate MRD in human cancer surgery patients, followed by additional surgical stress. Similar to the prophylactic model, we observed that AdDCT-vaccinated mice who received resection alone survived significantly longer than AdDCT-vaccinated mice with additional surgical stress (Fig 5b). Given the suppressive effects of surgery on pre-existing DCT-specific CD8^+^ T cell immunity, we administered preoperative cytokine immunotherapy in the form of IFNα treatment to rescue T cell function and improve survival (Fig 5c). We observed that the median survival of AdDCT vaccinated mice that underwent resection and nephrectomy was 17 days, while median survival was extended to 27 days in perioperative IFNα treated mice (Fig 5d). Next, we directly examined whether IFNα treatment in the context of AdDCT vaccination functions to improve DCT-specific T cell responses. Interestingly, preoperative IFNα therapy did not significantly impact cytokine secretion (IFNγ and TNFα) from DCT-specific CD8^+^ T cells (S4 Fig). Taken together, these results demonstrate that we can combine immunotherapy with major surgery to prolong survival in the postoperative period. However, other effector cells (such as NK cells) may play a role in addition to DCT-specific CD8^+^ T cells in mediating the beneficial effects of IFNα.

### Surgery-induced expansion of granulocytic MDSC impairs T cell cytokine secretion

**Fig 6:**
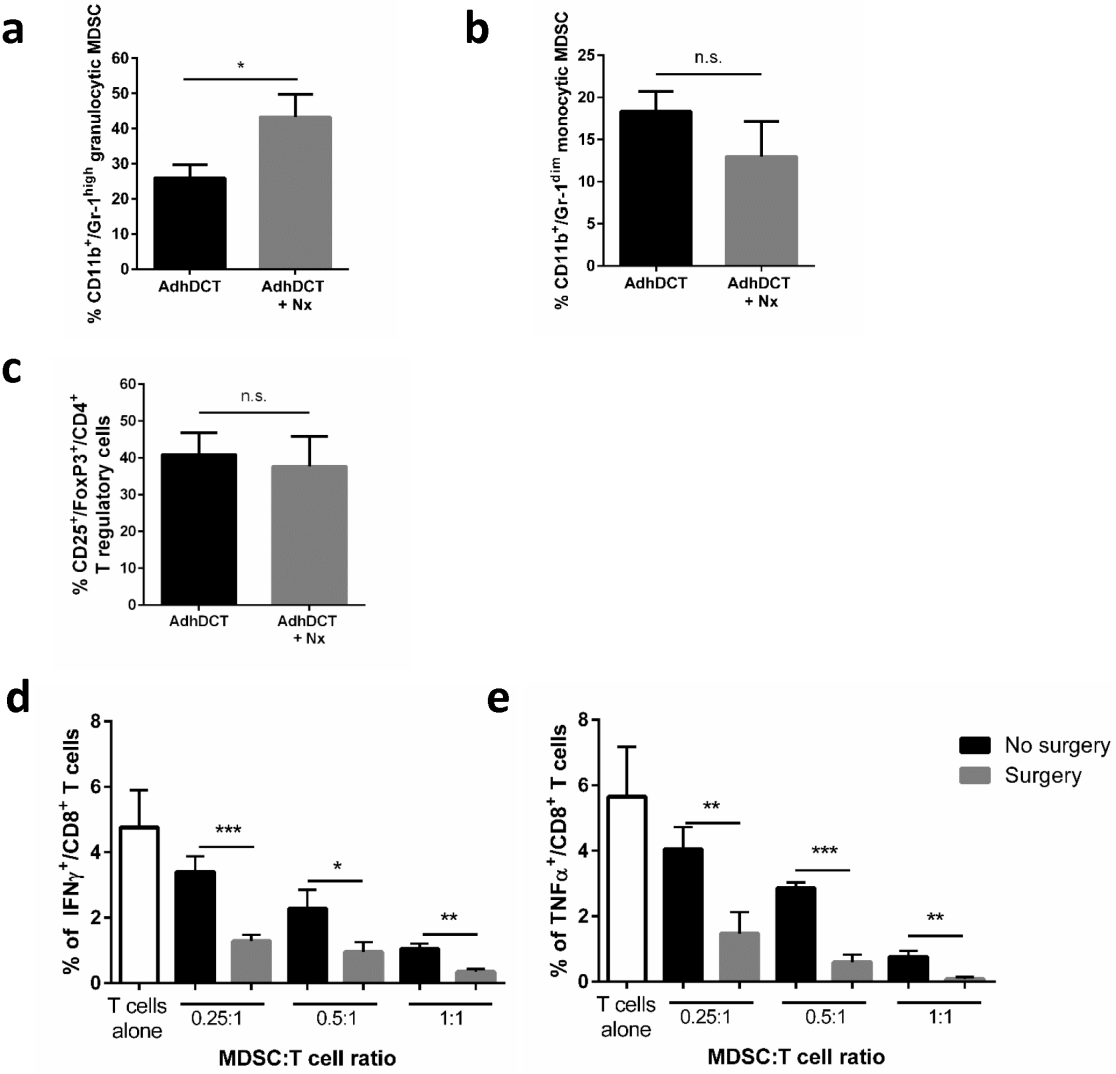
Accumulation of granulocytic myeloid derived suppressor cells following surgical stress impairs cytokine secretion by TAA-specific T cells. B6 mice were challenged with B16F10lacZ tumors, received AdDCT vaccination (day 7) and then underwent surgery as described above. At day 8, mice were sacrificed and underwent splenic MDSC were isolated for enumeration and functional assays. Percentage of **(a)** granulocytic MDSC, **(b)** monocytic MDSC and **(c)** T regulatory cells. Percentage of DCT-specific **(d)** IFNγ^+^ and **(e)** TNFα^+^ CD8^+^ T cells from control or vaccinated mice reacting to DCT_180-188_ peptide exposure following 4 days in culture with control or surgically stressed MDSC. N = 4-6/group. (*P<0.05, **P<0.01, ***P<0.001).

Lastly, we investigated the mechanisms of T cell suppression in tumor-bearing mice receiving vaccination and undergoing surgical stress. Myeloid Derived Suppressor Cells (MDSC) represent a population of immature myeloid cells that expand dramatically during tumor progression and impair adaptive immunity [25]. In mice there are two populations of MDSC defined by their relative expression of Gr1 and functional status [26, 27]. Specifically, we observed a significant increase in the proportion of spleen granulocytic MDSC (gMDSC) in surgically stressed and vaccinated mice (Fig 6a), but not monocytic MDSC (mMDSC) (Fig 6b) compared to vaccinated controls without surgery. In contrast, we did not observe any differences in spleen T regulatory (Treg) populations following vaccine and surgery (Fig 6c). To analyze the ability of surgery-derived gMDSC to suppress vaccine-activated T cell function, splenic gMDSC were isolated from control and surgically stressed mice and cocultured with T cells from Ad-DCT-vaccinated mice. In the presence of surgery-derived gMDSC, the production of IFNγ (Fig 6d) and TNFα (Fig 6e) by CD8^+^ DCT-specific T cells were significantly inhibited in response to DCT peptide. Additionally, we assessed whether preoperative IFNα treatment can reverse the accumulation of gMDSCs in spleen of mice following vaccination and surgery (S5 Fig). Upon closer examination, we found that preoperative IFNα therapy does not decrease the expansion of gMDSC in mice when compared to vaccinated mice undergoing surgery. We further show that gMDSC cell surface expression of the activation/maturation markers CD80/CD86 are not altered following preoperative IFNα suggesting that IFNα treatment does not directly impact gMDSC composition and function in the spleen.

## Discussion

Surgical resection is the mainstay of therapy for most solid malignancies, but despite complete resection, many patients harbour MRD and ultimately die of a recurrence [28]. Cancer vaccines are best suited to eradicate MRD [22, 23, 29], providing a strong rationale to combine surgery and immunotherapy in cancer clinical trials. However, a logically designed clinical trial of cancer vaccination must take into consideration the effect of surgical stress on TAA-specific T cell responses and the mechanisms responsible it.

In the present study, we demonstrated that the transient, but profound suppression of TAA-specific CD8^+^ T-cell immunity following major surgery promotes the development of cancer metastases and local recurrence in a mouse model of melanoma and cancer vaccination. Specifically, we incorporated the effects of a major surgical procedure by subjecting mice to a laparotomy and nephrectomy with AdDCT vaccination and observed that 100% of vaccinated and surgically stressed mice developed a tumor recurrence at a rate similar to mice that received PBS (Fig 1d and 1e). To definitively conclude that surgery was suppressing a T cell mediated anti-tumor immune response, we adoptively transferred T cells from surgically stressed vaccinated mice into naïve recipient mice, followed by a flank tumor challenge. We observed 90% protection of those mice that received donor T cells from no surgery vaccinated mice, while those that received T cells from surgically stressed vaccinated donors all developed progressive flank tumors and died (Fig 2d). We subsequently characterized the effect of major surgery on DCT-specific CD8^+^ T cell immunity and observed dramatically decreased IFNγ-, TNFα- and Granzyme B-secreting DCT-specific splenic CD8^+^ T cells (Fig 3a–3f) beginning 24 hours after surgery and lasting 7–10 days (S3 Fig). Moreover, we observed a significant reduction in global numbers of splenic CD8^+^ T cells following surgery (Fig 4a). This reduction of total CD8^+^ T cells is likely caused by a combination of reduced proliferation (Fig 4c) and migration away from the spleen to sites of trauma. We have previously observed that CFSE labelled whole splenocytes migrate preferentially to sites of surgical trauma following surgical stress [3], where the peritoneal cavity is the site of surgical trauma following abdominal nephrectomy.

To recover surgery-mediated immune defects, we administered IFNα in the preoperative period and observed that IFNα treatment significantly extends survival in surgically stressed mice (Fig 5c and 5d). While we hypothesize that IFNγ secretion by tumor-antigen specific CD8^+^ T cells contribute to the survival benefit of preoperative IFNα treated mice, our data clearly suggest that other effector cells, possibly NK or dendritic cells, play an important role in mediating this process as preoperative IFNα administration did not impact cytokine secretion when CD8^+^ T cells were isolated (S4 Fig). However, more work will be needed to clarify the relative roles of these individual cells types and the importance of their collective function in reducing metastasis.

Importantly, we provide mechanistic evidence that surgery expands a suppressive population of gMDSC that impair TAA-specific IFNγ and TNFα release by T cells following co-culture (Fig 6). These results clearly indicate that major surgery can significantly attenuate a pre-existing protective T cell immune response following cancer vaccination, which is mediated by gMDSC.

Even when splenocytes were non-specifically stimulated with phorbol myristate acetate (PMA) and ionomycin, both mitogens that activate the NFAT (nucelar factor of activated T cells) transcription factor in T lymphocytes [30], secretion of IFNγ was significantly attenuated in AdDCT-vaccinated and surgically stressed mice (S2 Fig). This, along with our tetramer analysis suggest that surgery-induced dysfunction of T cells is independent of the TCR (T-cell receptor) and could be due to other external factors. Generalized T cell dysfunction has been documented in patients following major surgery, including a significant decrease in CD4^+^ and CD8^+^ T cell numbers [31, 32] associated with increased apoptosis [33–35], and a severe defect in cytokine secretion [31, 36] and proliferation following non-specific *in vitro* stimulation. Our study also demonstrated a significant reduction in CD8^+^ T cell infiltration of tumors following surgical stress (Fig 4g) suggesting that T cell migration and tumor homing are also be impaired postoperatively.

Numerous studies have shown the immunosuppressive effects of MDSC on T cell function, proliferation [26, 37–39] and migration to tumors [40]. We have demonstrated that gMDSC not only accumulate after surgery but are more highly suppressive of antigen-specific T cell cytokine secretion (Fig 6d and 6e). This is consistent with studies demonstrating that trauma results in accumulation of highly suppressive MDSC that can impair global T cell proliferation and function [41–43]. The molecular mechanisms of MDSC accumulation following surgery are have not been clearly defined and represent ongoing objectives in our laboratory.

The immediate postoperative period provides an ideal environment for the cancer growth and metastases. Despite this, it remains a therapeutic window that is largely ignored in our current cancer treatment paradigm. Our findings have important therapeutic implications. First, native anti-tumor T cell immunity against cancer might be sufficient to eradicate micrometastatic disease and therefore identifying strategies to reverse postoperative adaptive anti-tumor T cell dysfunction following surgery could improve prognosis for all cancer surgery patients. Towards this end, a few clinical trials of perioperative low dose IFNα and IL-2 have been conducted with promising early clinical results [44–47]. Moreover, therapeutic cancer vaccines are emerging as potential therapies and they are best suited to eradicate MRD [22, 23, 29], providing a strong rationale to combine them with surgery. However, any such clinical trial would have to take into account the effects of surgical stress on MDSC mediated T cell dysfunction and consider preoperative immunostimulation strategies to protect the postoperative antitumor immune response.

## Supporting Information

S1 Fig Attenuation of pre-existing anti-tumor immunity by surgical stress in a CT26 colorectal cancer model. BALB/c mice were implanted sc with 1x10^6^ syngeneic CT26lacZ colorectal cancer cells. Following multiple iv treatments with oncolytic Vesicular Stomatitis Virus (VSV), mice underwent sham laparotomy (Sx) at day 18, leaving the primary tumor intact. At day 19, mice were challenged with 1x10^6^ secondary CT26lacZ cells on the opposite flank. Percentage of mice with secondary CT26lacZ tumor outgrowth 50 days post-challenge is shown, N = 4-5/group.
(PDF)

S2 Fig Reduction in the number of CD8^+^ T cells secreting IFNγ following surgery in response to non-specific stimulation with PMA and Ionomycin. B6 mice were challenged iv with 3x10^5^ of B16F10lacZ cells in order to establish syngeneic lung melanoma metastases. At day 7, mice received 1×10^7^ pfu AdDCT and then underwent surgery or no surgery. At day 8, mice were sacrificed and underwent spleen immune cell assessment. Percentage of PMA/Ionomycin stimulated IFNγ^+^, CD8^+^ T cells is shown. (*P<0.05, ***P<0.001).
(PDF)

S3 Fig Recovery of T cell functionality between post-operative day (POD) 7 and POD 28 and improved survival at POD 28. **(a)** B6 mice were challenged iv with 3x10^5^ of B16F10lacZ cells in order to establish syngeneic lung melanoma metastases. At day 7, mice received 1×10^7^ pfu AdDCT and then underwent surgery or no surgery. **(b)** Percentage of DCT-specific IFNγ^+^/CD8^+^ T cells reacting to DCT_180-188_ peptide exposure at 1, 3, 7, and 28-days post-surgery. N = 4-5/group. **(c)** Survival of treated B16F10lacZ tumor-bearing mice challenged 28 days post-surgery shown in Kaplan-Meier curves. Percentage of living mice is indicated. N = 7-8/group, (*P<0.05, ***P<0.001).
(PDF)

S4 Fig Preoperative IFNα treatment following AdDCT vaccination and surgery does not improve DCT-specific T cell responses. B6 mice received 1×10^7^ pfu AdDCT at day 0. On day 7, the mice underwent surgery or no surgery. Preoperative treatment was initiated at day 3 with 1 high dose (10,000 IU/mouse) and at days 4 through 6 with 3 low doses (1000 IU/mouse) of recombinant mIFNα. Percentage of **(a)** DCT-specific IFNγ^+^/CD8^+^ T cells and **(b)** DCT-specific TNFα^+^/CD8^+^ T cells reacting to DCT_180-188_ peptide exposure, PMA/Ionomycin or no stimulation at 1 day post-surgery. N = 5-7/group. (*P<0.05, **P<0.01).
(PDF)

S5 Fig Preoperative IFNα treatment following AdDCT vaccination and surgery does not reverse the accumulation of spleen gMDSCs. B6 mice received 1×10^7^ pfu AdDCT at day 0. On day 7, the mice underwent surgery or no surgery. Preoperative treatment was initiated at day 3 with 1 high dose (10,000 IU/mouse) and at days 4 through 6 with 3 low doses (1000 IU/mouse) of recombinant mIFNα. Percentage of **(a)** granulocytic MDSC (CD11b^+^/Gr1^high^) and **(b)** CD80^+^/CD86^+^ gMDSC (CD11b^+^/Gr1^high^) at 1 day post-surgery. N = 5-7/group. (*P<0.05, ***P<0.001).
(PDF)

S1 Table Animal wellness program of the Animal Care and Veterinary Services of the University of Ottawa. Mice are wellnessed daily following surgery. Score key: M1, mild; M2, moderate; M3, severe. POD, postoperative day; BW, bodyweight; Abd Nx, abdominal nephrectomy.
(PDF)
