# Correction: Surgical Stress Abrogates Pre-Existing Protective T Cell Mediated Anti-Tumor Immunity Leading to Postoperative Cancer Recurrence

**DOI:** 10.1371/journal.pone.0159471

**Published:** 2016-07-18

**Authors:** Abhirami A. Ananth, Lee-Hwa Tai, Casey Lansdell, Almohanad A. Alkayyal, Katherine E. Baxter, Leonard Angka, Jiqing Zhang, Christiano Tanese de Souza, Kyle B Stephenson, Kelley Parato, Jonathan L Bramson, John C Bell, Brian D Lichty, Rebecca C Auer

“Cancer Research Society, Operating Grant” was omitted from the Financial Disclosure of the published article. The correct Financial Disclosure is: This work was supported by Terry Fox Research Institute, New Investigator Award (RCA); Canadian Institutes of Health Research Fellowship (LHT); Canadian Cancer Society Research Institute, Innovation and Innovation to Impact Grant (RCA), and Cancer Research Society, Operating Grant (RCA).

## References

[1] Ananth AA, Tai L-H, Lansdell C, Alkayyal AA, Baxter KE, Angka L, et al (2016) Surgical Stress Abrogates Pre-Existing Protective T Cell Mediated Anti-Tumor Immunity Leading to Postoperative Cancer Recurrence. PLoS ONE 11(5): e0155947 doi:10.1371/journal.pone.0155947 27196057 10.1371/journal.pone.0155947 PMC4873120

