# Supplementary figures and images for: Surgical Stress Abrogates Pre-Existing Protective T Cell Mediated Anti-Tumor Immunity Leading to Postoperative Cancer Recurrence
Source: PLoS One. 2016 May 19;11(5):e0155947. doi: 10.1371/journal.pone.0155947 (PMC4873120; doi:10.1371/journal.pone.0155947)

# Supplemental Figure 1

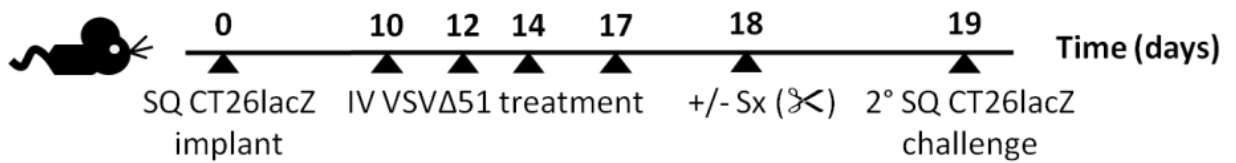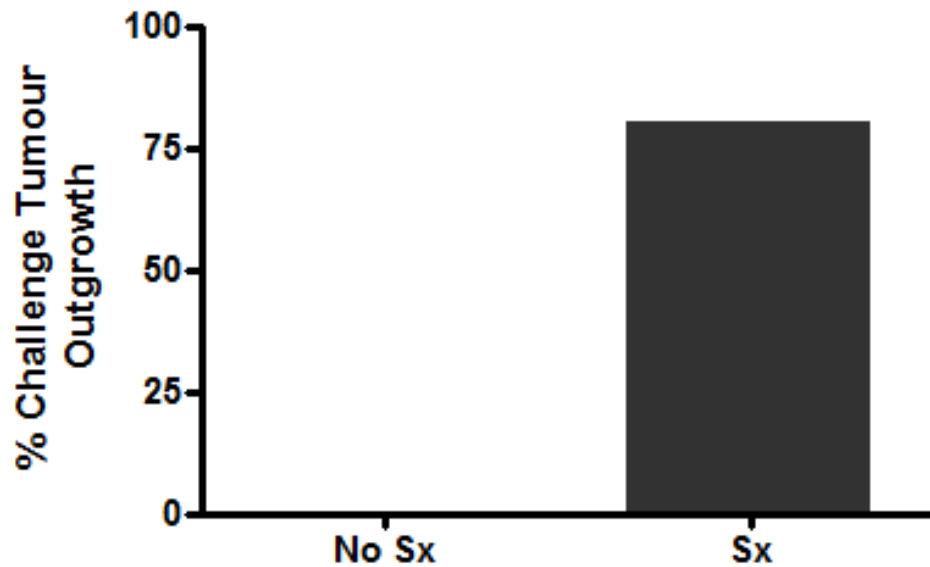

Supplement: S1 Fig — BALB/c mice were implanted sc with 1x10^6^ syngeneic CT26lacZ colorectal cancer cells. Following multiple iv treatments with oncolytic Vesicular Stomatitis Virus (VSV), mice underwent sham laparotomy (Sx) at day 18, leaving the primary tumor intact. At day 19, mice were challenged with 1x10^6^ secondary CT26lacZ cells on the opposite flank. Percentage of mice with secondary CT26lacZ tumor outgrowth 50 days post-challenge is shown, N = 4-5/group. (PDF) [file pone.0155947.s001.pdf]

# Supplemental Figure 2

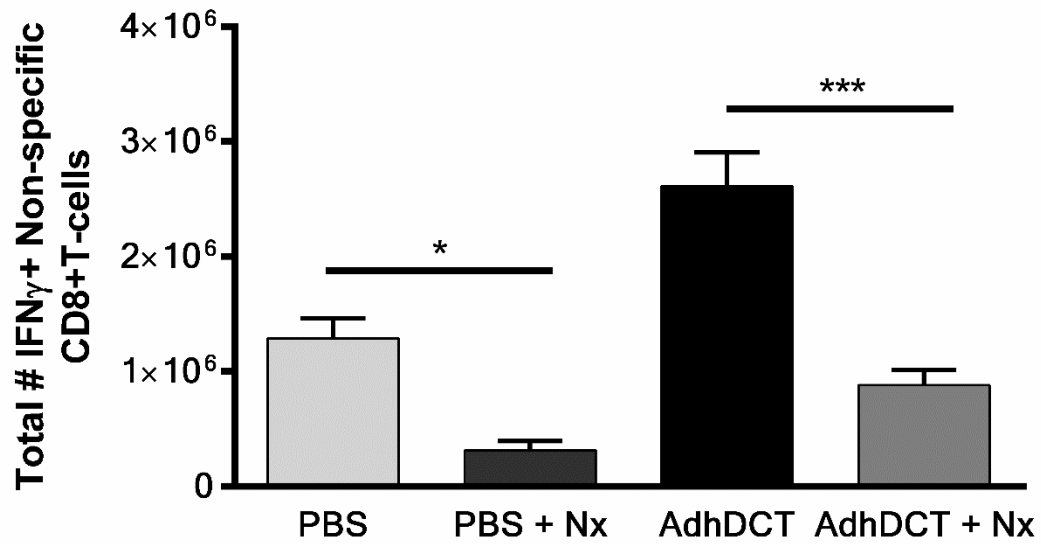

Supplement: S2 Fig — B6 mice were challenged iv with 3x10^5^ of B16F10lacZ cells in order to establish syngeneic lung melanoma metastases. At day 7, mice received 1×10^7^ pfu AdDCT and then underwent surgery or no surgery. At day 8, mice were sacrificed and underwent spleen immune cell assessment. Percentage of PMA/Ionomycin stimulated IFNγ+, CD8+ T cells is shown. (*P<0.05, ***P<0.001). (PDF) [file pone.0155947.s002.pdf]

# Supplemental Figure 3

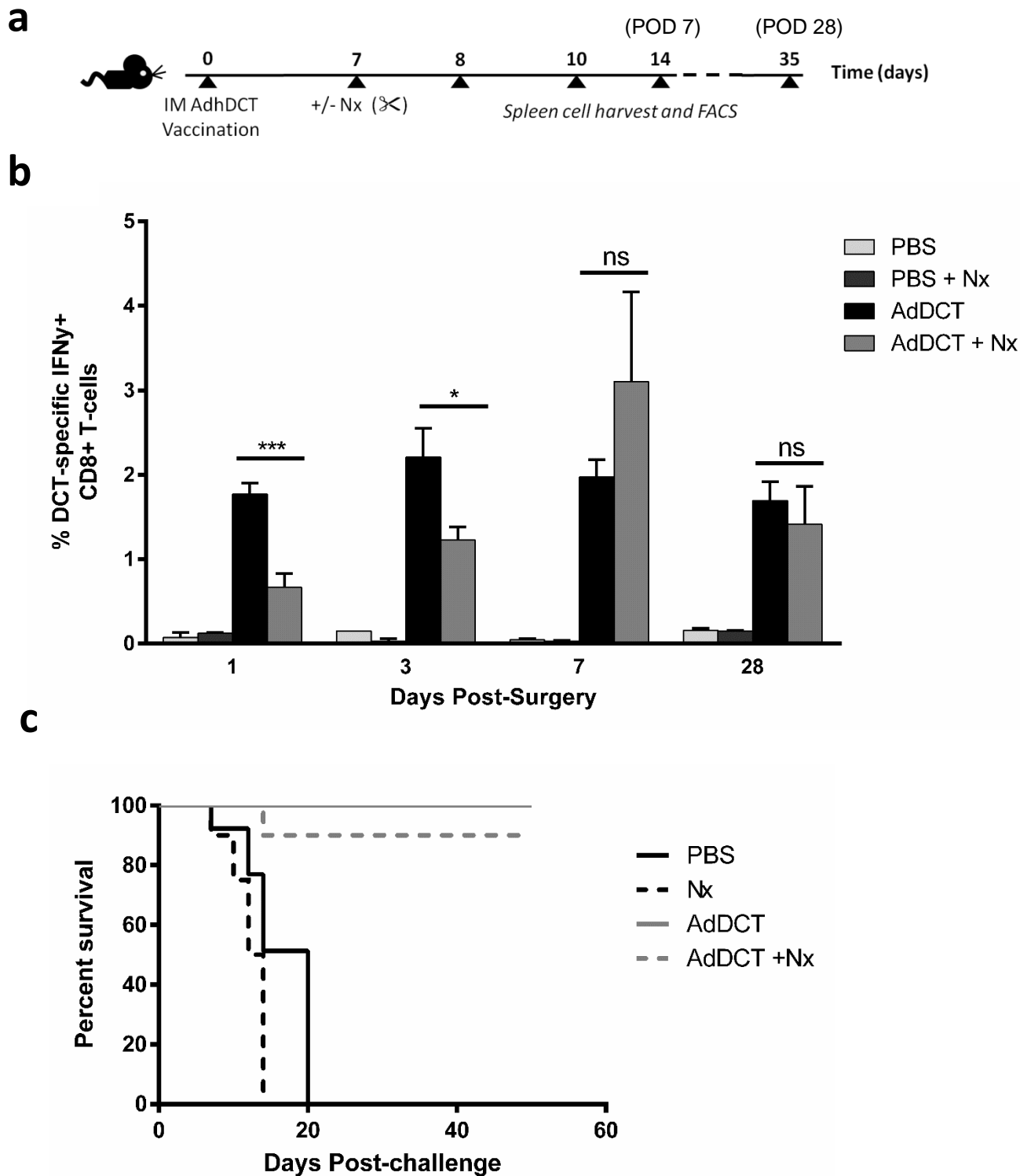

Supplement: S3 Fig — (a) B6 mice were challenged iv with 3x10^5^ of B16F10lacZ cells in order to establish syngeneic lung melanoma metastases. At day 7, mice received 1×10^7^ pfu AdDCT and then underwent surgery or no surgery. (b) Percentage of DCT-specific IFNγ+/CD8+ T cells reacting to DCT180-188 peptide exposure at 1, 3, 7, and 28-days post-surgery. N = 4-5/group. (c) Survival of treated B16F10lacZ tumor-bearing mice challenged 28 days post-surgery shown in Kaplan-Meier curves. Percentage of living mice is indicated. N = 7-8/group, (*P<0.05, ***P<0.001). (PDF) [file pone.0155947.s003.pdf]

# Supplemental Figure 4

**a**

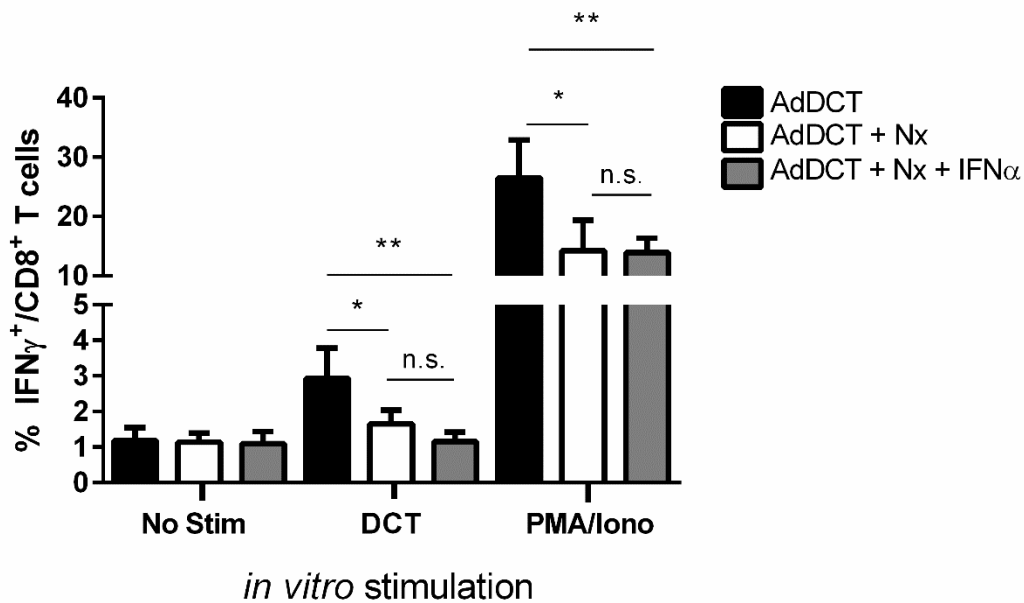

**b**

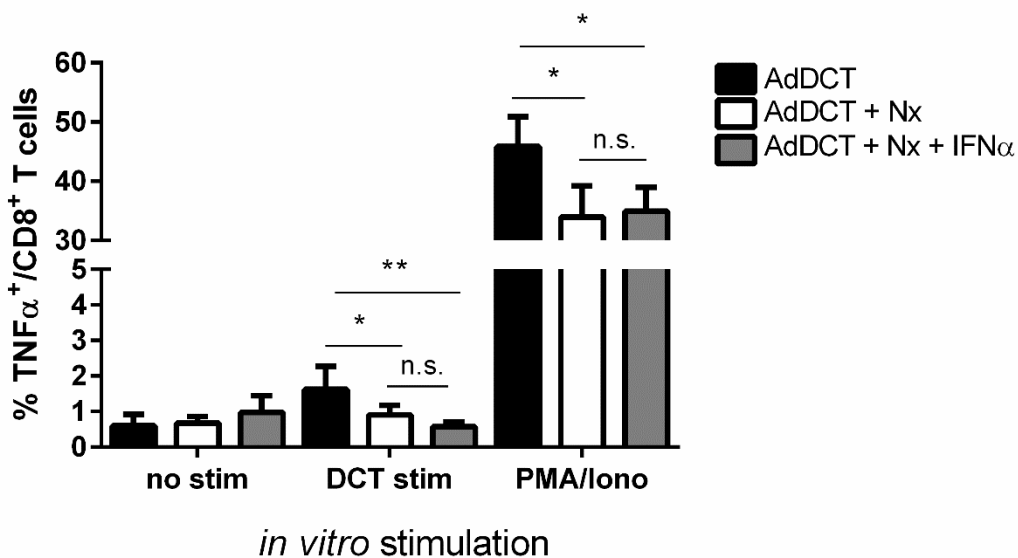

Supplement: S4 Fig — B6 mice received 1×10^7^ pfu AdDCT at day 0. On day 7, the mice underwent surgery or no surgery. Preoperative treatment was initiated at day 3 with 1 high dose (10,000 IU/mouse) and at days 4 through 6 with 3 low doses (1000 IU/mouse) of recombinant mIFNα. Percentage of (a) DCT-specific IFNγ+/CD8+ T cells and (b) DCT-specific TNFα+/CD8+ T cells reacting to DCT180-188 peptide exposure, PMA/Ionomycin or no stimulation at 1 day post-surgery. N = 5-7/group. (*P<0.05, **P<0.01). (PDF) [file pone.0155947.s004.pdf]

## Supplemental Figure 5

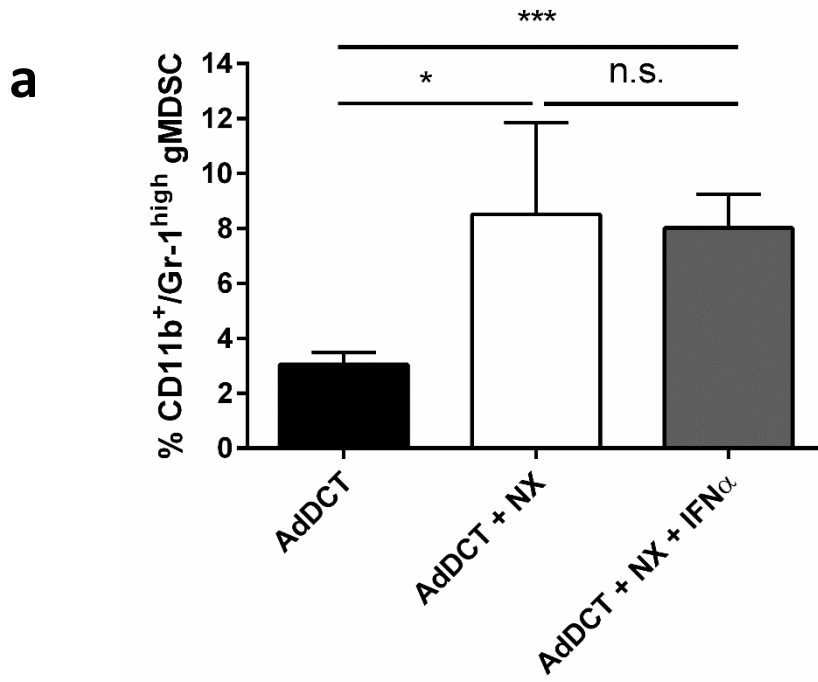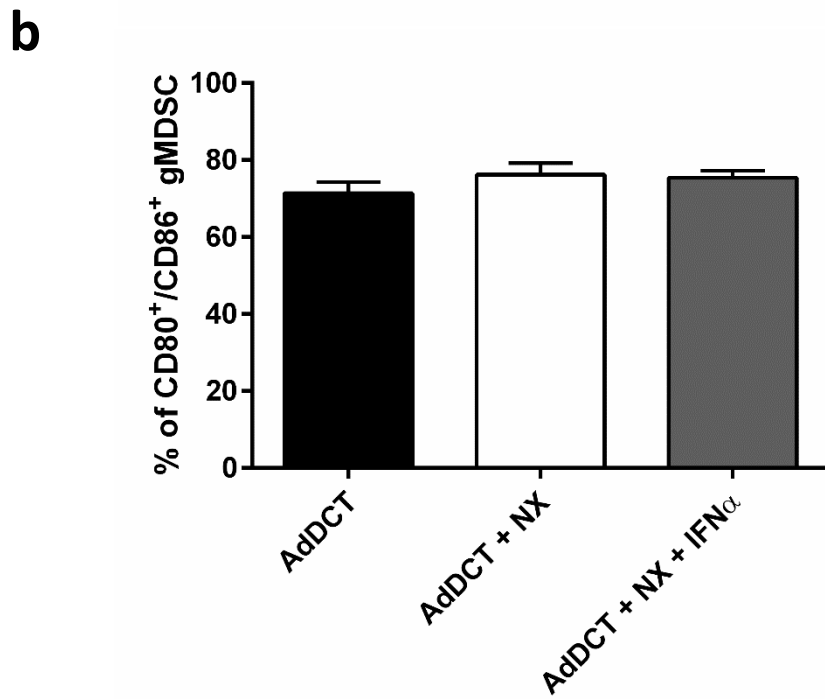

Supplement: S5 Fig — B6 mice received 1×10^7^ pfu AdDCT at day 0. On day 7, the mice underwent surgery or no surgery. Preoperative treatment was initiated at day 3 with 1 high dose (10,000 IU/mouse) and at days 4 through 6 with 3 low doses (1000 IU/mouse) of recombinant mIFNα. Percentage of (a) granulocytic MDSC (CD11b+/Gr1high) and (b) CD80+/CD86+ gMDSC (CD11b+/Gr1high) at 1 day post-surgery. N = 5-7/group. (*P<0.05, ***P<0.001). (PDF) [file pone.0155947.s005.pdf]
