# Supplementary material for: Surgical Stress Abrogates Pre-Existing Protective T Cell Mediated Anti-Tumor Immunity Leading to Postoperative Cancer Recurrence
Source: PLoS One. 2016 May 19;11(5):e0155947. doi: 10.1371/journal.pone.0155947 (PMC4873120; doi:10.1371/journal.pone.0155947)
Supplement: S1 Table — Mice are wellnessed daily following surgery. Score key: M1, mild; M2, moderate; M3, severe. POD, postoperative day; BW, bodyweight; Abd Nx, abdominal nephrectomy. (PDF) [file pone.0155947.s006.pdf]

Supplemental Table 1

Animal ID	Procedure	Baseline BW	Attitude	Piloerection	Facial Grimace Score	Hydration
1	abd nx	19.7	-	-	-	-
2	abd nx	22.5	-	-	-	-
3	abd nx	18.5	-	-	-	-
4	abd nx	19.2	-	-	-	-
5	abd nx	20.2	-	-	-	-

Animal ID	Procedure	POD1 BW	Attitude	Piloerection	Facial Grimace Score	Hydration
1	abd nx	19.5	M1	M2	M1	M1
2	abd nx	22	M2	M2	M1	M2
3	abd nx	18.1	M1	M2	M1	M1
4	abd nx	19	M1	M1	M1	M1
5	abd nx	20	M1	M1	M1	M1

Animal ID	Procedure	POD2 BW	Attitude	Piloerection	Facial Grimace Score	Hydration
1	abd nx	19.2	M2	M2	M2	M2
2	abd nx	21.9	M2	M2	M2	M2
3	abd nx	17.9	M2	M2	M2	M2
4	abd nx	18.6	M1	M2	M2	M2
5	abd nx	19.6	M1	M2	M2	M2

Animal ID	Procedure	POD3 BW	Attitude	Piloerection	Facial Grimace Score	Hydration
1	abd nx	18.6	M2	M2	M2	M3
2	abd nx	21.5	M2	M2	M2	M2
3	abd nx	17.6	M2	M3	M2	M3
4	abd nx	18	M2	M3	M2	M2
5	abd nx	19.1	M2	M2	M2	M2
